# 3-(4-Methyl­phen­yl)-2-thioxo-1,3-thia­zolidin-4-one

**DOI:** 10.1107/S1600536810029569

**Published:** 2010-07-31

**Authors:** Durre Shahwar, M. Nawaz Tahir, Naeem Ahmad, Muhammad Asam Raza, Saherish Aslam

**Affiliations:** a Department of Chemistry, Government College University, Lahore, Pakistan; b Department of Physics, University of Sargodha, Sargodha, Pakistan

## Abstract

In the title compound, C_10_H_9_NOS_2_, the toluene group and the 2-thioxo-1,3-thia­zolidin-4-one unit are planar with r.m.s. deviations of 0.0082 and 0.0136 Å, respectively. The dihedral angle between them is 71.20 (9)°. In the crystal, the mol­ecules are stabilized through inter­molecular C—H⋯O contacts, forming polymeric sheets extending parallel to the (0
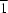
1) plane. C—H⋯π contacts also occur.

## Related literature

For related structures and the preparation, see: Shahwar *et al.* (2009*a* ▶,*b* ▶).
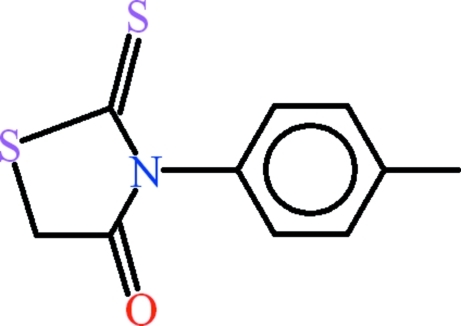


## Experimental

### 

#### Crystal data

C_10_H_9_NOS_2_
*M* *_r_* = 223.30
Monoclinic, 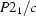

*a* = 14.5885 (18) Å
*b* = 5.5766 (6) Å
*c* = 12.9910 (15) Å
β = 100.603 (6)°
*V* = 1038.8 (2) Å^3^
*Z* = 4
Mo *K*α radiation
μ = 0.48 mm^−1^
*T* = 296 K
0.30 × 0.20 × 0.20 mm

#### Data collection

Bruker Kappa APEXII CCD diffractometer
Absorption correction: multi-scan (*SADABS*; Bruker, 2005 ▶) *T*_min_ = 0.939, *T*_max_ = 0.950
7426 measured reflections
1871 independent reflections
1371 reflections with *I* > 2σ(*I*)
*R*_int_ = 0.051

#### Refinement

*R*[*F* ^2^ > 2σ(*F* ^2^)] = 0.059
*wR*(*F* ^2^) = 0.191
*S* = 1.07
1871 reflections
128 parameters
H-atom parameters constrained
Δρ_max_ = 0.45 e Å^−3^
Δρ_min_ = −0.31 e Å^−3^

### 

Data collection: *APEX2* (Bruker, 2009 ▶); cell refinement: *SAINT* (Bruker, 2009 ▶); data reduction: *SAINT*; program(s) used to solve structure: *SHELXS97* (Sheldrick, 2008 ▶); program(s) used to refine structure: *SHELXL97* (Sheldrick, 2008 ▶); molecular graphics: *ORTEP-3 for Windows* (Farrugia, 1997 ▶) and *PLATON* (Spek, 2009 ▶); software used to prepare material for publication: *WinGX* (Farrugia, 1999 ▶) and *PLATON*.

## Supplementary Material

Crystal structure: contains datablocks global, I. DOI: 10.1107/S1600536810029569/si2279sup1.cif

Structure factors: contains datablocks I. DOI: 10.1107/S1600536810029569/si2279Isup2.hkl

Additional supplementary materials:  crystallographic information; 3D view; checkCIF report

## Figures and Tables

**Table 1:** Hydrogen-bond geometry (Å, °) *Cg*2 is the centroid of the C1–C6 benzene ring.

*D*—H⋯*A*	*D*—H	H⋯*A*	*D*⋯*A*	*D*—H⋯*A*
C2—H2⋯O1^i^	0.93	2.45	3.360 (5)	167
C5—H5⋯O1^ii^	0.93	2.51	3.432 (5)	169
C9—H9*B*⋯*Cg*2^iii^	0.97	2.71	3.565 (4)	147

Symmetry codes: (i) 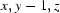; (ii) 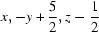; (iii) 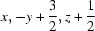.

**Fig. 1.**
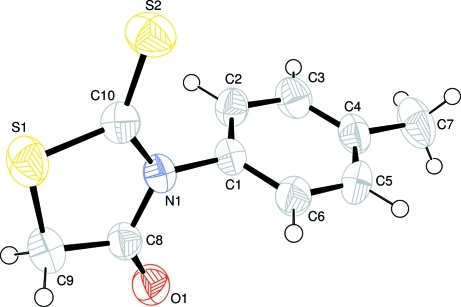
View of the title compound with the atom numbering scheme. The thermal ellipsoids are drawn at the 50% probability level. H-atoms are shown by small circles of arbitrary radii.

**Fig. 2.**
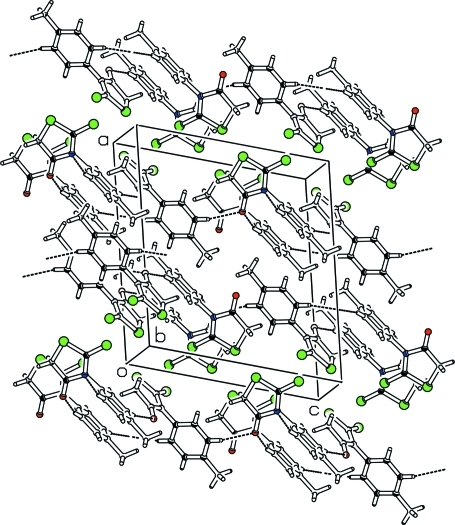
The partial packing (PLATON; Spek, 2009) which shows that molecules form polymeric sheets extending parallel to (0 1 1).

## supplementary crystallographic information

### Comment

In continuation to synthesize various derivatives of 2-thioxo-1,3-thiazolidin-4-one, the title compound (I, Fig. 1) is being reported.

The crystal structure of (II) 3-(2-methylphenyl)-2-thioxo-1,3-thiazolidin-4-one (Shahwar *et al.*, 2009*a*) and (III) 3-(3-methylphenyl)-2-thioxo-1,3-thiazolidin-4-one (Shahwar *et al.*, 2009*b*) have been published which differ from (I) due to the position of methyl group.

In (I), the toluene group A (C1—C7) and group B (N1/C8—C10/S1/S2/O1) of 2-thioxo-1,3-thiazolidin-4-one moiety are planar with maximum r. m. s. deviations of 0.0082 and 0.0136 Å, respectively. The dihedral angle between A/B is 71.20 (9)°. This value is different from 84.44 (9)° and 83.30 (3)° as observed in (II) and (III), respectively. The molecules are stabilized in the form polymeric sheets due to C—H···O type of intermolecular H-bondings and C—H···π contacts (Table 1). The polymeric sheets extend parallel to the (0 1 1) plane (Fig. 2).

### Experimental

The title compound has been prepared by the method described in (Shahwar *et al.*, 2009*a*) and (Shahwar *et al.*, 2009*b*).

### Refinement

All H-atoms were positioned geometrically (C–H = 0.93–0.97 Å) and refined as riding with *U*_iso_(H) = *xU*_eq_(C), where *x* = 1.2 for aryl and *x* = 1.5 for methyl H-atoms.

### Crystal data

**Table:**

C_10_H_9_NOS_2_	*F*(000) = 464
*M**_r_* = 223.30	*D*_x_ = 1.428 Mg m^−^^3^
Monoclinic, *P*2_1_/*c*	Mo *K*α radiation, λ = 0.71073 Å
Hall symbol: -P 2ybc	Cell parameters from 1371 reflections
*a* = 14.5885 (18) Å	θ = 3.3–25.2°
*b* = 5.5766 (6) Å	µ = 0.48 mm^−^^1^
*c* = 12.9910 (15) Å	*T* = 296 K
β = 100.603 (6)°	Prism, light yellow
*V* = 1038.8 (2) Å^3^	0.30 × 0.20 × 0.20 mm
*Z* = 4	

### Data collection

**Table:**

Bruker Kappa APEXII CCD diffractometer	1871 independent reflections
Radiation source: fine-focus sealed tube	1371 reflections with *I* > 2σ(*I*)
graphite	*R*_int_ = 0.051
Detector resolution: 8.10 pixels mm^-1^	θ_max_ = 25.2°, θ_min_ = 3.2°
ω scans	*h* = −17→17
Absorption correction: multi-scan (*SADABS*; Bruker, 2005)	*k* = −6→6
*T*_min_ = 0.939, *T*_max_ = 0.950	*l* = −14→15
7426 measured reflections	

### Refinement

**Table:**

Refinement on *F*^2^	Primary atom site location: structure-invariant direct methods
Least-squares matrix: full	Secondary atom site location: difference Fourier map
*R*[*F*^2^ > 2σ(*F*^2^)] = 0.059	Hydrogen site location: inferred from neighbouring sites
*wR*(*F*^2^) = 0.191	H-atom parameters constrained
*S* = 1.07	*w* = 1/[σ^2^(*F*_o_^2^) + (0.1099*P*)^2^ + 0.6595*P*] where *P* = (*F*_o_^2^ + 2*F*_c_^2^)/3
1871 reflections	(Δ/σ)_max_ < 0.001
128 parameters	Δρ_max_ = 0.45 e Å^−^^3^
0 restraints	Δρ_min_ = −0.31 e Å^−^^3^

### Special details

**Table:**

Geometry. Bond distances, angles *etc*. have been calculated using the rounded fractional coordinates. All su's are estimated from the variances of the (full) variance-covariance matrix. The cell e.s.d.'s are taken into account in the estimation of distances, angles and torsion angles
Refinement. Refinement of *F*^2^ against ALL reflections. The weighted *R*-factor *wR* and goodness of fit *S* are based on *F*^2^, conventional *R*-factors *R* are based on *F*, with *F* set to zero for negative *F*^2^. The threshold expression of *F*^2^ > σ(*F*^2^) is used only for calculating *R*-factors(gt) *etc*. and is not relevant to the choice of reflections for refinement. *R*-factors based on *F*^2^ are statistically about twice as large as those based on *F*, and *R*- factors based on ALL data will be even larger.

### Fractional atomic coordinates and isotropic or equivalent isotropic displacement parameters (Å^2^)

**Table:**

	*x*	*y*	*z*	*U*_iso_*/*U*_eq_	
S1	0.07809 (7)	0.7565 (2)	0.48054 (8)	0.0640 (4)	
S2	0.08797 (7)	0.4645 (2)	0.29429 (9)	0.0645 (4)	
O1	0.29145 (18)	1.1330 (5)	0.45318 (19)	0.0576 (9)	
N1	0.20548 (18)	0.8215 (5)	0.3708 (2)	0.0419 (8)	
C1	0.2640 (2)	0.7927 (6)	0.2936 (2)	0.0422 (10)	
C2	0.3229 (2)	0.5995 (7)	0.2997 (3)	0.0486 (11)	
C3	0.3830 (3)	0.5829 (7)	0.2290 (3)	0.0559 (12)	
C4	0.3849 (3)	0.7554 (7)	0.1539 (3)	0.0522 (11)	
C5	0.3251 (3)	0.9453 (8)	0.1489 (3)	0.0638 (14)	
C6	0.2640 (3)	0.9667 (7)	0.2192 (3)	0.0561 (14)	
C7	0.4528 (3)	0.7357 (9)	0.0792 (3)	0.0774 (18)	
C8	0.2270 (2)	0.9984 (6)	0.4471 (3)	0.0455 (11)	
C9	0.1587 (3)	0.9972 (8)	0.5211 (3)	0.0559 (12)	
C10	0.1289 (2)	0.6787 (6)	0.3748 (3)	0.0470 (11)	
H2	0.32242	0.48179	0.35029	0.0584*	
H3	0.42296	0.45210	0.23248	0.0672*	
H5	0.32517	1.06212	0.09782	0.0767*	
H6	0.22378	1.09710	0.21555	0.0676*	
H7A	0.48304	0.88736	0.07508	0.1162*	
H7B	0.41973	0.69135	0.01101	0.1162*	
H7C	0.49876	0.61574	0.10415	0.1162*	
H9A	0.12591	1.14909	0.51789	0.0672*	
H9B	0.19110	0.97175	0.59244	0.0672*	

### Atomic displacement parameters (Å^2^)

**Table:**

	*U*^11^	*U*^22^	*U*^33^	*U*^12^	*U*^13^	*U*^23^
S1	0.0506 (6)	0.0820 (8)	0.0656 (7)	−0.0038 (5)	0.0268 (5)	−0.0086 (5)
S2	0.0524 (6)	0.0690 (8)	0.0715 (8)	−0.0092 (5)	0.0097 (5)	−0.0155 (5)
O1	0.0639 (16)	0.0610 (17)	0.0485 (15)	−0.0104 (14)	0.0122 (12)	−0.0037 (12)
N1	0.0403 (14)	0.0497 (16)	0.0369 (14)	0.0036 (12)	0.0106 (11)	−0.0002 (12)
C1	0.0431 (18)	0.050 (2)	0.0345 (16)	0.0003 (14)	0.0098 (13)	−0.0013 (14)
C2	0.051 (2)	0.052 (2)	0.0429 (19)	0.0044 (16)	0.0093 (15)	0.0068 (16)
C3	0.051 (2)	0.066 (2)	0.052 (2)	0.0110 (18)	0.0132 (16)	−0.0024 (19)
C4	0.049 (2)	0.070 (2)	0.0395 (18)	−0.0142 (18)	0.0133 (15)	−0.0076 (17)
C5	0.089 (3)	0.063 (2)	0.044 (2)	−0.003 (2)	0.024 (2)	0.0133 (18)
C6	0.075 (3)	0.049 (2)	0.047 (2)	0.0102 (18)	0.0185 (18)	0.0057 (17)
C7	0.060 (3)	0.123 (4)	0.055 (2)	−0.022 (2)	0.026 (2)	−0.019 (2)
C8	0.052 (2)	0.0459 (19)	0.0391 (18)	0.0040 (16)	0.0099 (15)	0.0013 (14)
C9	0.056 (2)	0.069 (2)	0.045 (2)	0.0045 (18)	0.0150 (16)	−0.0037 (17)
C10	0.0400 (18)	0.053 (2)	0.048 (2)	0.0015 (15)	0.0080 (14)	0.0010 (15)

### Geometric parameters (Å, °)

**Table:**

S1—C9	1.798 (5)	C4—C7	1.513 (6)
S1—C10	1.732 (4)	C5—C6	1.394 (6)
S2—C10	1.627 (4)	C8—C9	1.507 (5)
O1—C8	1.194 (4)	C2—H2	0.9300
N1—C1	1.441 (4)	C3—H3	0.9300
N1—C8	1.393 (4)	C5—H5	0.9300
N1—C10	1.381 (4)	C6—H6	0.9300
C1—C2	1.371 (5)	C7—H7A	0.9600
C1—C6	1.370 (5)	C7—H7B	0.9600
C2—C3	1.385 (5)	C7—H7C	0.9600
C3—C4	1.374 (5)	C9—H9A	0.9700
C4—C5	1.366 (6)	C9—H9B	0.9700
			
S1···N1	2.568 (3)	C6···H9B^vii^	3.0300
S1···S1^i^	3.7490 (16)	C7···H3^viii^	3.0200
S1···S1^ii^	3.6396 (16)	C8···H6	3.0500
S2···C2	3.497 (3)	C8···H7B^v^	2.9800
S2···C8^iii^	3.651 (4)	C10···H2	3.1000
S1···H9A^ii^	3.0300	H2···O1^iii^	2.4500
S2···H6^iii^	3.1500	H2···C10	3.1000
O1···C2^iv^	3.360 (5)	H3···H7C	2.3500
O1···C6	3.132 (5)	H3···C7^ix^	3.0200
O1···C7^v^	3.321 (5)	H5···H7A	2.5700
O1···H2^iv^	2.4500	H5···O1^x^	2.5100
O1···H5^vi^	2.5100	H6···S2^iv^	3.1500
O1···H7B^v^	2.6100	H6···C8	3.0500
N1···S1	2.568 (3)	H7A···H5	2.5700
C2···S2	3.497 (3)	H7A···H7A^xi^	2.4500
C2···O1^iii^	3.360 (5)	H7B···O1^vii^	2.6100
C4···C8^vii^	3.500 (5)	H7B···C2^vii^	3.0800
C6···O1	3.132 (5)	H7B···C8^vii^	2.9800
C7···O1^vii^	3.321 (5)	H7C···H3	2.3500
C8···S2^iv^	3.651 (4)	H9A···S1^ii^	3.0300
C8···C4^v^	3.500 (5)	H9B···C1^v^	3.0200
C1···H9B^vii^	3.0200	H9B···C2^v^	3.0300
C2···H9B^vii^	3.0300	H9B···C3^v^	3.0400
C2···H7B^v^	3.0800	H9B···C4^v^	3.0700
C3···H9B^vii^	3.0400	H9B···C5^v^	3.0400
C4···H9B^vii^	3.0700	H9B···C6^v^	3.0300
C5···H9B^vii^	3.0400		
			
C9—S1—C10	93.85 (18)	S2—C10—N1	127.1 (3)
C1—N1—C8	119.3 (3)	C1—C2—H2	121.00
C1—N1—C10	123.3 (3)	C3—C2—H2	121.00
C8—N1—C10	117.4 (3)	C2—C3—H3	119.00
N1—C1—C2	119.5 (3)	C4—C3—H3	119.00
N1—C1—C6	119.3 (3)	C4—C5—H5	120.00
C2—C1—C6	121.1 (3)	C6—C5—H5	119.00
C1—C2—C3	118.7 (3)	C1—C6—H6	121.00
C2—C3—C4	121.5 (4)	C5—C6—H6	120.00
C3—C4—C5	118.7 (4)	C4—C7—H7A	109.00
C3—C4—C7	120.4 (4)	C4—C7—H7B	109.00
C5—C4—C7	120.9 (4)	C4—C7—H7C	109.00
C4—C5—C6	121.0 (4)	H7A—C7—H7B	109.00
C1—C6—C5	119.0 (4)	H7A—C7—H7C	109.00
O1—C8—N1	124.3 (3)	H7B—C7—H7C	109.00
O1—C8—C9	124.5 (3)	S1—C9—H9A	110.00
N1—C8—C9	111.2 (3)	S1—C9—H9B	110.00
S1—C9—C8	106.9 (3)	C8—C9—H9A	110.00
S1—C10—S2	122.26 (19)	C8—C9—H9B	110.00
S1—C10—N1	110.7 (2)	H9A—C9—H9B	109.00
			
C9—S1—C10—N1	1.8 (3)	C10—N1—C8—C9	−0.3 (4)
C10—S1—C9—C8	−1.9 (3)	N1—C1—C6—C5	176.0 (3)
C9—S1—C10—S2	−178.1 (3)	C6—C1—C2—C3	0.1 (5)
C10—N1—C1—C2	−72.1 (4)	N1—C1—C2—C3	−175.8 (3)
C8—N1—C10—S2	178.7 (3)	C2—C1—C6—C5	0.0 (5)
C8—N1—C1—C6	−69.3 (4)	C1—C2—C3—C4	0.3 (6)
C10—N1—C1—C6	111.9 (4)	C2—C3—C4—C7	178.6 (4)
C8—N1—C1—C2	106.7 (4)	C2—C3—C4—C5	−0.7 (6)
C1—N1—C10—S1	177.6 (2)	C3—C4—C5—C6	0.9 (6)
C8—N1—C10—S1	−1.2 (4)	C7—C4—C5—C6	−178.5 (4)
C1—N1—C10—S2	−2.5 (5)	C4—C5—C6—C1	−0.5 (6)
C1—N1—C8—O1	0.7 (5)	O1—C8—C9—S1	−178.3 (3)
C10—N1—C8—O1	179.5 (3)	N1—C8—C9—S1	1.5 (4)
C1—N1—C8—C9	−179.1 (3)		

Symmetry codes: (i) −*x*, −*y*+1, −*z*+1; (ii) −*x*, −*y*+2, −*z*+1; (iii) *x*, *y*−1, *z*; (iv) *x*, *y*+1, *z*; (v) *x*, −*y*+3/2, *z*+1/2; (vi) *x*, −*y*+5/2, *z*+1/2; (vii) *x*, −*y*+3/2, *z*−1/2; (viii) −*x*+1, *y*+1/2, −*z*+1/2; (ix) −*x*+1, *y*−1/2, −*z*+1/2; (x) *x*, −*y*+5/2, *z*−1/2; (xi) −*x*+1, −*y*+2, −*z*.

### Hydrogen-bond geometry (Å, °)

**Table:**

Cg2 is the centroid of the C1–C6 benzene ring.

**Table:**

*D*—H···*A*	*D*—H	H···*A*	*D*···*A*	*D*—H···*A*
C2—H2···O1^iii^	0.93	2.45	3.360 (5)	167
C5—H5···O1^x^	0.93	2.51	3.432 (5)	169
C9—H9B···Cg2^v^	0.97	2.71	3.565 (4)	147

Symmetry codes: (iii) *x*, *y*−1, *z*; (x) *x*, −*y*+5/2, *z*−1/2; (v) *x*, −*y*+3/2, *z*+1/2.
